# Healthy dietary patterns, typical healthy foods and risk of endometrial cancer: a systematic review and meta-analysis of observational studies

**DOI:** 10.3389/fonc.2026.1901081

**Published:** 2026-08-12

**Authors:** Jiner Chen, Yiyi Xu, Ke Zhao

**Affiliations:** Obstetrics and Gynecology, Zhuji Sixth People’s Hospital, Zhuji, Zhejiang, China

**Keywords:** endometrial cancer, healthy dietary patterns, meta-analysis, systematic review, typical healthy foods

## Abstract

**Background:**

Previous observational studies have investigated the associations between adherence to healthy dietary patterns and endometrial cancer risk; however, pooled quantitative evidence synthesizing healthy dietary patterns and typical healthy foods remains limited. Therefore, we conducted this systematic review and meta-analysis to quantitatively synthesize available evidence on healthy dietary patterns, typical healthy foods and endometrial cancer risk.

**Methods:**

Studies published up to February 2026 were systematically identified using PubMed, ISI Web of Science, Embase and China National Knowledge Infrastructure (CNKI) databases. Random- effects or fixed-effects models were used to calculate pooled relative risks (RRs) with corresponding 95% confidence intervals (CIs) where appropriate. Heterogeneity across studies was quantified using Cochran’s Q test and the I^2^ statistics.

**Results:**

Thirty-five articles with a total of 533,828 participants and 17,758 endometrial cancer cases were included in our analysis. Compared with the lowest intake of healthy dietary patterns, the highest intake was associated with a 22% lower risk of endometrial cancer (RR = 0.78; 95% CI: 0.65- 0.93, *P* = 0.006).Additionally, the highest intakes of total vegetables (RR = 0.83;95%CI:0.75-0.92, *P* = 0.001) and whole grains (RR = 0.90; 95%CI:0.84-0.98, *P* = 0.012) were significantly associated with reduced risk of endometrial cancer. Comparatively, high intakes of fruits and fish showed no statistically significant association with endometrial cancer risk (*P*>0.05).

**Conclusions:**

This systematic review and meta-analysis showed that high intakes of healthy dietary patterns, vegetables and whole grains were significantly with a lower risk of endometrial cancer. Further studies, particularly well-designed prospective cohort studies, are needed to confirm these findings.

## Introduction

Endometrial cancer is the most common gynecological malignancy in Western countries, and its incidence is rising rapidly worldwide (1). According to GLOBOCAN 2020, endometrial cancer is the sixth most commonly diagnosed cancer among women, with an estimated 400,000 new cases, accounting for approximately 2.2% of all cancer cases globally (2). In the United States, the annual number of endometrial cancer cases is projected to double, reaching 122,000 by 2030 (3). Thus, prioritizing the identification of modifiable risk factors for endometrial cancer is of critical importance. Established risk factors for endometrial cancer include genetic predisposition, advanced age, nulliparity, prolonged lifetime exposure to estrogen, early menarche, late menopause, tamoxifen use, family history of cancer, metabolic syndrome, and obesity (4). In addition to these factors, diet as a modifiable lifestyle factor, has been shown to play an important role in endometrial cancer prevention (5).

Over the past few decades, research on the association between diet and endometrial cancer risk has primarily focused on specific food groups or individual nutrients (6–8). Notably, a previous meta- analysis of observational studies found that consumption of vegetables and fruits was significantly associated with a reduced risk of endometrial cancer (9). The researchers suggested that vegetables and fruits may exert beneficial effects through their antioxidant and detoxifying properties, modulation of the immune system, and regulation of hormone concentrations. However, findings from published studies have not been entirely consistent. For example, Yeh et al. observed that higher intakes of vegetables and vegetable-related nutrients were associated with a decreased risk of endometrial cancer (6). In contrast, a large prospective cohort study reported no significant association between higher consumption of vegetables or fruits and endometrial cancer risk (10). Furthermore, given the complexity of dietary composition and the potential interactions among food components (5), studies focusing on single foods or nutrients may have limited power to capture the overall effect of diet on chronic non-communicable diseases, including endometrial cancer. Consequently, dietary pattern analysis has become a more recognizable approach, because it accounts for the synergistic and cumulative effects of various food groups and nutrients and may better inform nutritional recommendations (11).

The relationship between overall dietary patterns and endometrial cancer risk has been a concern for years (12). To date, numerous observational studies have investigated the associations between various healthy dietary patterns and endometrial cancer risk (13–23). Indeed, these studies have shown that healthy dietary patterns, such as the Mediterranean diet, the Dietary Approaches to Stop Hypertension (DASH) diet, and prudent dietary pattern, are typically characterized by high intakes of fruits, vegetables, whole grains, nuts, and legumes (12). However, epidemiological evidence regarding the association between endometrial cancer risk and healthy dietary patterns remains inconsistent. Some studies have shown the significant protective effects (14, 15, 18, 20– 21), whereas others have reported no such associations (13, 16, 17, 19, 22, 23). Notably, two previous meta-analyses have explored the associations between dietary patterns and endometrial cancer risk (5, 24). In 2017, Si et al. synthesized available evidence from observational studies and concluded that healthy dietary patterns were associated with a decreased risk of endometrial cancer (5). However, that study had notable limitations in identification of dietary patterns, particularly in treating single food items as proxies for overall dietary pattern. For example, the authors considered total vegetables intake from the studies by Yeh et al. (4) and McCullough et al. (10) as representative of healthy dietary pattern, and pooled these results with those from Dalvi et al. (13), which reported an inverse association between plant-based pattern and endometrial cancer. Similarly, another previous meta-analysis of five observational studies showed that healthy dietary pattern was associated with a lower risk of endometrial cancer (24). However, it is important to note that this meta-analysis systematically searched for studies published prior to March 2016. Since then, several new studies (15, 18, 19, 21) have been published, allowing for more comprehensive and statistically robust synthesis of the evidence. Given these knowledge gaps in the existing literature, this systematic review and meta-analysis aimed to summarize observational studies published up to February 2026, and to comprehensively assess the associations between healthy dietary pattern, typical healthy foods and the risk of endometrial cancer.

## Methods

This updated systematic review and meta-analysis was conducted in accordance with the Preferred Reporting Items for Systematic Reviews and Meta-Analyses (PRISMA) 2020 guidelines (25).

### Literature search strategy

The literature published up to February 2026 was searched for relevant articles, using four electronic databases, including PubMed, ISI Web of Science, Embase, and CNKI, without language restrictions. The following search terms were used: (“endometrial cancer” [all fields] OR “endometrial neoplasms” [all fields] OR “endometrial tumor” [all fields] OR “endometrial carcinoma” [all fields]) AND (“diet” [all fields] OR “food” [all fields] OR “dietary pattern” [all fields] OR “food pattern” [all fields]). The detailed search strategy for each database can be found in **Supplementary Table 1**. Additionally, the reference lists of retrieved articles and published reviews were manually searched to identify potentially eligible studies. Gray literature and unpublished studies were not included.

### Selection criteria

Two authors (J.-E.C. and K.Z.) independently screened and crosschecked each article from the literature search, and a third author (Y.-Y.X.) was consulted to resolve any discrepancies. The titles and abstracts of all retrieved articles were screened to identify published studies examining the associations between dietary patterns, food groups and risk of endometrial cancer. The inclusion criteria were as follows (1): observational studies, including case-control, nested case-control or cohort studies; (2) published in English or Chinese; (3) considered healthy dietary patterns or typical healthy foods as the exposure of interest; (4) reported endometrial cancer as the outcome of interest; (5) provided adjusted estimates of RRs (e.g. ORs, RRs or HRs) and 95% CIs for the associations of healthy dietary patterns, healthy foods, and endometrial cancer risk; (6) if the data in original study lacked sufficient detail, the corresponding author was contacted by email for additional information. The exclusion criteria were as follows: (1) non-observational studies (e.g. reviews, editorials, case reports, letters, commentaries, or conference abstract); (2) published in languages other than English or Chinese; (3) not performed in humans (animal studies, cell culture, or *in vitro* experiments); (4) studies with insufficient data. When multiple articles were published from the same study, we selected the newest publication with the largest number of cases. The Participant, Exposure, Comparison, Outcome, and Study design (PECOS) criteria for inclusion and exclusion of studies is shown in **Supplementary Table 2**.

### Data extraction

Two aforementioned authors (J.-E.C. and K.Z.) independently extracted the following information from each eligible study: first author’s last name, publication year, country where study was performed, study design, age range/mean age for study participants, total number of cases and controls or participants, methods used to identify healthy dietary patterns, adjustment or matching for potential confounders in multivariate analyses, and the effect estimates (e.g. ORs, RRs or HRs) with corresponding 95% CIs for the highest versus lowest categories of healthy dietary patterns or typical healthy foods. Disagreements regarding data extraction were resolved by consensus or through discussion with the corresponding author (Y.-Y.X).

### Quality assessment

The Newcastle-Ottawa Scale (NOS), designed for non-randomized studies, was used to assess the quality of each selected study (26). The NOS consists of eight items across three main domains: selection (maximum 4 stars), comparability (maximum 2 stars), and assessment of outcome/exposure of interest for cohort/case-control studies (maximum 3 stars), with a total score of 9 stars. In our analysis, studies with a NOS score of 7 stars or higher were considered high quality (27). Two authors (J.-E.C and K.Z.) independently performed the quality assessment, and disagreements were resolved by discussion or consultation with the corresponding author (Y.-Y.X.).

### Statistical analysis

We used RRs and 95%CIs as the risk estimate for the main analysis. For case-control studies, ORs were converted into RRs using the following formula: RR=OR/[(1-P_0_)+(P_0_*OR)], in which P_0_ shows the incidence of endometrial cancer in the non-exposed group (28). Besides, HRs were assumed to approximate RRs (29). Due to the expected heterogeneity among the included studies, a random-effects model (DerSimonian and Laird method) was used to calculate pooled RRs and 95% CIs for the associations between endometrial cancer risk and the highest versus lowest intake categories of healthy dietary patterns and typical healthy foods. Between-study heterogeneity was quantified using Cochran’s Q test and the I^2^ statistics, in which *P* value of Q-test ≤ 0.10 or I^2^ ≥50% was defined as significant heterogeneity (30). When heterogeneity was low (Q-test *P*>0.10 or I^2^<50%), a fixed-effects model was used. In the presence of significant heterogeneity, subgroup and sensitivity analyses were carried out to further explore potential sources of heterogeneity. Subgroup analyses was stratified by study design, methods used to determine healthy dietary patterns, country, study quality, comparison, sample and case sizes for healthy dietary patterns; and by study design, country, study quality, sample size and comparison for typical healthy foods. Sensitivity analyses were performed by sequentially excluding one study at a time to assess the robustness of the results. Publication bias was assessed by visual inspection of funnel plots, formal testing by the Egger’s regression asymmetry and Begg’s rank correlation tests (31). When evidence of publication bias was detected, the trim-and-fill method of Duval and Tweedie was utilized to adjust the pooled RRs (32). All statistical analyses were performed using STATA version 18.0 (StataCorp, College Station, Texas, USA), with a two-tailed *P-*value<0.05 considered statistically significant.

## Results

### Overview of included studies

**Figure 1:**
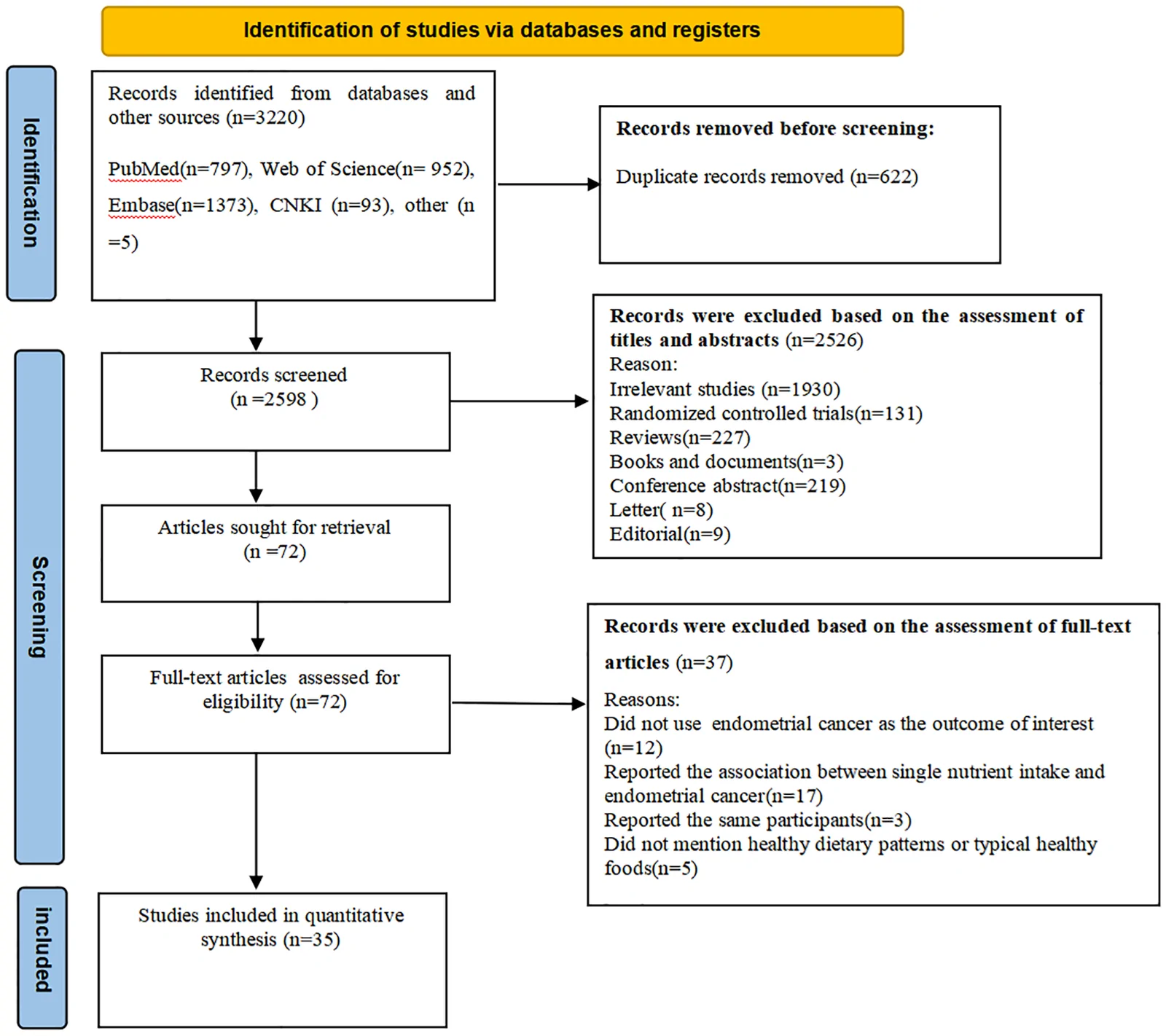
The flowchart of the study selection process.

**Figure 1** shows the flowchart of the study selection process. Initially, we identified 3,220 potentially relevant articles by databases searches and manual screening of reference lists After eliminating 622 duplicates, 2,598 articles remained for title and abstract screening, of which 2,526 articles were excluded because they did not examine the associations between dietary patterns or typical healthy foods and endometrial cancer risk. The remaining 72 articles underwent full-text review, of which 37 were excluded for the following reasons: endometrial cancer was not the outcome of interest (n=12); the study reported associations between single nutrient intake and endometrial cancer (17); reported the same participants (n=3); did not mention healthy dietary patterns or typical healthy foods (n=5). Finally, thirty-five articles (6–8, 10, 13–23, 33–52) met the eligibility criteria and were included in this updated systematic review and meta-analysis. Among included articles, ten articles reported on the association between healthy dietary patterns and endometrial cancer risk (13–23); twenty articles reported on total vegetable intake (6–8, 10, 18, 21, 23, 35, 36, 38, 40–47, 49, 51); eighteen articles on fruit intake (6–8, 10, 21, 23, 36–38, 40–45, 47, 48, 51); six articles reported on whole grains intake (7, 8, 23, 33, 39, 40), and eight articles on fish intake (7, 18, 34, 36, 42, 46, 50, 52) in relation to endometrial cancer. .

### Study characteristics

The characteristics of the included studies on the associations between healthy dietary patterns, typical healthy foods and endometrial cancer risk are summarized in **Table 1**. Thirty-five articles with a total of 533,828 participants and 17,758 endometrial cancer cases were included in the analyses. Most of the included studies were case-control studies (6, 8, 13–16, 18, 20, 21, 23, 35–37, 40–48, 50–52) and ten were cohort studies (7, 10, 17, 19, 22, 33, 34, 38, 39, 49). All included studies were published between 1986 and 2026. The follow-up time for cohort studies ranged from 9.3 to 20.4 years. Fourteen of the included studies were performed in the United States (6, 8, 10, 13, 17, 19, 20, 23, 34, 38, 39, 41, 42, 44), four in Italy (16, 18, 21, 36), three in Sweden (48–50), two in China (47, 52), two in Japan (37, 46), two in Canada (14, 22), two in Greece (43, 51), one in Denmark (33), one in the UK (7), one in France (40), one in Mexico (45), one in Iran (15), and one study in Italy and Sweden (35). The age of participants ranged from ages 18 to above. Seven of included studies derived dietary patterns using a posteriori method (13, 14, 16, 17, 20–23), and three studies used *a priori* dietary scores (15, 18, 19). Twenty-eight of included studies used FFQs to collect dietary data (6, 7, 10, 13–23, 33–36, 38, 39, 41, 43–48, 52), six studies used questionnaires (8, 37, 40, 42, 49, 50), and one study used 24-h dietary recall (51). The main adjustment variables included age, energy intake, education, age at menarche, oral contraceptive use, menopausal status, parity, physical activity, family history of cancer, alcohol drinking, history of hypertension, smoking, diabetes and body mass index. Overall, twenty-nine articles were classified as high quality (6–8, 10, 13, 14, 17–23, 33–39, 41, 42, 45–50, 52), and the remaining six were classified as medium quality (15, 16, 40, 43– 44, 51).

**Table 1:** Characteristics of included studies on the associations between healthy dietary patterns, typical healthy foods and endometrial cancer risk (–2026).

Author publication year	Location	Study design	Total number of subjects	Age	Method used to identify dietary pattern	Adjustment or matched for in analyses	RR/OR(95%CI) for highest versus lowest category
Yeh et al., 2009 (6)	United States	Case-control	541 cases 541 controls	27-96y	NA	Age, BMI (continuous variable), exogenous estrogen use, smoking, total menstrual months, and total energy.	Total vegetables: OR 0.51; 95%CI:0.34-0.75; total fruits: OR 1.1; 95% CI: 0.74-1.62.
Dunneram et al., 2019 (7)	UK	Cohort	32,289 (294 cases)	35-69y	NA	Age, ethanol intake, duration of breastfeeding, physical activity, smoking, social class, menopausal status, history of diabetes and history of hypertension.	Whole grain: HR = 0.92; 95% CI:0.84-1.01;Total vegetables: HR=0.93; 95%CI: 0.80-1.08; Total fruits: HR = 0.90; 95%CI: 0.79-1.03.
Goodman et al., 1997 (8)	USA	Case-control	332 cases 511 controls	18-84y	NA	Pregnancy history, birth control pill use, estrogen use, history of diabetes mellitus, Quetelet’s index (kg/m^2^), and total calories	Vegetables: OR: 0.51; 95%CI: 0.29-0.90;Fruit: OR: 0.48; 95%CI:0.28-0.80; Whole grain: OR: 0.48; 95%CI: 0.29- 0.79.
McCullough et al., 2007 (10)	USA	Cohort	41,400 (435 cases)	50-74y	NA	Age (years), age at menarche, age at Menopause, number of livebirths and age at first birth, hormone replacement therapy (HRT), past combined HRT, other HRT/unknown type and unknown use, or missing data),cigarette smoking, recreational physical activity in quintiles, total energy intake, and body mass index (weight (kg)/height (m)^2^	Vegetables: RR = 1.21; 95% CI: 0.89-1.65; fruits: RR = 1.24; 95%CI: 0.90-1.70.
Dalvi et al., 2007 (13)	USA	Case-control	488 cases 461 controls	35-79y	Posteriori	Age, race/ethnicity, age at menarche, OC use, parity, average daily caloric intake, average weekly physical activity, and the joint effects of menopausal status, HT use, and BMI.	Plant-based pattern: OR: 1.2; 95%CI:0.72-2.0
Biel et al., 2011 (14)	Canada	Case-control	506 cases 981 controls	30-79y	Posteriori	Age (years), total energy intake (kcal), BMI (*<*25, 25- 29.9, *>*30), age at menarche (year), parity (0, 1-2, *>*2 pregnancies, ≥20 week gestation), hypertension history (never vs. ever), hormone contraception use (never vs. ever), alcohol intake (0 drinks vs. *<*1 drink, ≥1 drink/day).	Healthier plants pattern: OR:0.70;95%CI:0.50-0.98.
Etesami et al.,2026 (15)	Iran	Case-control	136 cases 272 controls	40-79y	Priori	Age, waist circumference, physical activity, educational status, comorbidities, age at menarche, family history of endometrial cancer, menopausal status, use of oral contraceptive pills (OCPs) and hormone replacement therapy (HRT), vitamin D supplementation, and total energy intake.	Healthy eating index 2020:OR:0.17;95%CI: 0.09- 0.30.
Bravi et al., 2015 (16)	Italy	Case-control	454 cases 908 controls	18-80y	Posteriori	Age, study center, period of interview, education, body mass index, history of diabetes, age at menarche, menopausal status, parity, oral contraceptive use, hormone replacement therapy use, and all the five factors simultaneously.	Vitamins and fiber: OR: 0.96;95%CI:0.67-1.37.
Canchola et al., 2015 (17)	USA	Cohort	75,093 (937 cases)	≥18y	Posteriori	Race and its interaction with time-dependent age, age at menarche, gravidity and age at last pregnancy and its interaction with time-dependent age, oral contraceptive use, physical activity, smoking status, height, caloric intake, the other four dietary patterns, and the following time-dependent exposures: body mass index, menopausal status/hormone therapy use, and their interaction; age was the time metric, and the model was stratified by age at baseline.	Plant-based:RR:0.91; 95%CI: 0.72-1.15
Filomeni et al., 2015 (18)	Italy	Case-control	1411 cases 3668 controls	≥18 y	Priori	Age, study center, year of interview, education, tobacco smoking, body mass index, age at menopause, age at menarche, parity, oral contraceptive use, hormone-replacement therapy use, history of hypertension, diabetes and total energy intake.	Mediterranean diet: OR:0.43; 95%CI: 0.34- 0.56; Vegetables: OR: 0.64; 95%CI:0.55-0.75; Fish: OR: 1.00; 95%CI: 0.86 -1.17.
George et al., 2015 (19)	USA	Cohort	84,415 (1,392 cases)	50-79y	Prior	Age, energy intake, ethnicity, education, MET-h/week leisure time physical activity, diabetes status, postmenopausal hormone replacement therapy use, oral contraceptive use, age at first birth, participant in Observational Study, participant in HT trial, participant in DM trial.	HEI: HR1.11;95%CI:0.93-1.33AHEI: HR 0.98; 95% CI: 0.82- 1.17; aMED: HR 0.98; 95% CI:0.82-1.17; DASH diet: HR 1.00; 95%CI:0.84-1.19
McCann et al., 2001 (20)	USA	Case-control	232 cases 639 controls	40-85y	Posteriori	Energy, age, education, body mass index, diabetes, pack-years cigarette smoking, hypertension, age at menarche, oral contraceptive use, menopause status, parity, and menopausal estrogen use.	Healthy dietary pattern: OR 0.55; 95%CI: 0.35- 0.84.
Ricceri et al., 2017 (21)	Italy	Case-control	297 cases 307 controls	40-74y	Posteriori	Age, parity, menopausal status, hormone replacement therapy use, oral contraceptive use, body mass index, age at menarche, physical activity, education, smoking status, and total energy intake	Mediterranean diet: OR 0.51; 95%CI: 0.28- 0.92; Fruit: OR 0.55; 95% CI: 0.28-1.06; Vegetables: OR 0.34; 95%CI: 0.17-0.68.
Willemsen et al., 2022 (22)	Canada	Cohort	16,313 (138 cases)	35-69y	Posteriori	Age, sex, BMI, energy intake, smoking status, physical activity.	Prudent dietary pattern: HR: 0.79; 95% CI: 0.48- 1.29.
Chandran et al., 2010 (23)	USA	Case-control	424 cases 398 controls	≥21y	NA	Matched the patient on age (within 5y) and race (black or white).	HEI-2005: OR0.83; 95% CI: 0.52-1.34; total vegetables: OR 0.93; 95% CI:0.59-1.48. total fruits: OR 0.87; 95%CI: 0.55- 1.39.
Aarestrup et al.,2012 (33)	Denmark	Cohort	29,875 (217 cases)	50-64y	NA	Baseline values of menopausal status (post or pre), use of hormone replacement therapy (HRT) (never, former, present), type of HRT (unopposed estrogen, combination, other), parity (number of births, age at first pregnancy, nulliparity), body mass index (kg/m^2^), smoking(present, former, never), energy intake (kJ/day).	Total whole grain: RR1.08; 95%CI: 0.71 -1.65.
Arem et al., 2013 (34)	USA	Cohort	72,796 (1,486 cases)	50-71y	NA	Age, body mass index, smoking status, continuous total energy intake, other meat intake, age at menarche, age at first child’s birth, parity, age at menopause, HT use (never, ever), oral contraceptive use (never, ever), diabetes (yes, no) and physical activity.	Total fish: HR1.10; 95%CI: 0.93-1.29.
Bosetti et al., 2012 (35)	Italy/Sweden	Case-control	367 cases 798 controls	≥18y	NA	Sex (when appropriate), age, study center, year of interview, education, body mass index, alcohol drinking, tobacco smoking, and total energy intake.	Cruciferous vegetables: OR:0.93;95%CI:0.68-1.27
Bravi et al., 2009 (36)	Italy	Case-control	454 cases 908 controls	18-80y	NA	Age, center, year of interview, education, total energy intake, BMI, history of diabetes, age at menarche, parity, OC use, HRT, and menopausal status	All vegetables: OR 0.59; 95%CI:0.37-0.94.All fruits: OR 0.83; 95% CI: 0.55-1.24.
Hirose et al.,1996 (37)	Japan	Case-control	145 cases 26,751controls	≥18y	NA	Age, salty food, fatty food or total calories,	Fruit: OR1.97; 95%CI: 1.37- 2.82.
Kabat et al.,2010 (38)	USA	Cohort	112,088 (1,142 cases)	50-71y	NA	Age at baseline, race/ethnicity, education, age at menarche, parity, hormone therapy (ever, never), age at menopause, body mass index, smoking, frequency of vigorous physical activity, total fat intake, and caloric intake.	Total vegetables: HR = 1.09;95%CI:0.90-1.1.33;total fruits: HR = 1.30; 95% CI: 1.04-1.61
Kasum et al.,2001 (39)	USA	Cohort	23,014 (382 cases)	55-69y	NA	Age, kilocalories, education, body mass index, smoking, vitamin use, fruit and vegetable intake, red meat intake, refined grain intake, total fat, saturated fat, age at menarche, age at menopause, number of live births, and hormone use.	Whole grains: HR:0.89; 95% CI: 0.61-1.29.
La Vecchia et al., 1986 (40)	France	Case-control	206 cases 206 controls	≥18y	NA	Age and body mass index.	Green vegetables: RR 0.24; 95%CI: 0.13- 0.45; Fresh fruit: RR 0.57; 95% CI:0.33-0.99; Whole grains: RR0.59; 95% CI: 0.29-1.21.
Littman et al., 2001 (41)	USA	Case-control	679 cases 944 controls	45-74y	NA	Age in 5-year categories, county of residence (King, Pierce, Snohomish), total energy intake, unopposed estrogen use, and smoking.	Fruit: OR0.67;95%CI:0.47-0.95; Vegetables: OR 0.69; 95% CI: 0.48-1.0; Grains: OR 0.75; 95% CI: 0.50-1.2.
McCann et al., 2000 (42)	USA	Case-control	232 cases 639 controls	40-85y	NA	Age, education, BMI, diabetes, hypertension, pack-years cigarette smoking, age at menarche, parity, oral contraceptive use, menopause status, and postmenopausal estrogen use.	Total fruit:OR0.9; 95% CI:0.9-1.7;total vegetable: OR0.5; 95% CI:0.3-0.9; fish: OR 0.9; 95%CI:0.50- 1.5.
Petridou et al., 2002 (43)	Greece	Case-control	84 cases 84 controls	≥18y	NA	Age, education (in 6-year increments, ordinal), body mass index (BMI) before onset of symptoms (in 2-kg/m^2^ increments, ordinal), pregnancies andabortions (3 categories: nulliparity, pregnancies with a history of abortions, and pregnancies without a history of abortions), and total energy intake (in 1 SD increment, ordinal).	Fruit: OR:1.03;95%CI:0.74- 1.43; vegetables: OR1.25; 95%CI:0.89-1.74.
Potischman et al., 1993 (44)	United States	Case-control	399 cases 296 controls	20-74y	NA	Age-group, BMI, ever estrogen usage, ever oral- contraceptive usage, number of births, current smoking, education, total calories.	Fruit: OR:1.1;95%CI:0.6-1.9;vegetables: OR1.0; 95% CI: 0.6-1.6.
Salazar-Martinez et al., 2005 (45)	Mexico	Case-control	85 cases 668 controls	22-79y	NA	Age (5 years category and continuous), total energy intake, number of live births, body mass index, physical activity, and diabetes.	Fruit: OR0.88;95%CI:0.43-1.79; Green leafy vegetables: OR 1.11; 95% CI: 0.56-2.21.
Takayama et al., 2013 (46)	Japan	Case-control	161 cases 380 controls	22-79y	NA	Body mass index, diabetes history and hypertension history.	Vegetables: OR 0.45; 95%CI: 0.25-0.81; Fish: OR 0.53; 95% CI: 0.30- 0.94.
Tao et al.,2005 (47)	China	Case-control	832 cases 846 controls	30-69y	NA	Age, education, menopausal status, years of menstruation, first-degree family history of breast, colorectal, endometrial cancer, OC use, number of pregnancies, history of diabetes, BMI, total meat And fish intake and caloric intake.	Total vegetables: OR 0.69; 95%CI:0.50-0.96; fruits: OR 0.97; 95%CI: 0.72- 1.31.
Terry et al.,2002a (48)	Sweden	Case-control	709 cases 2888 controls	50-74y	NA	Age, body mass index, smoking, physical activity, prevalence of diabetes (yes, no), fatty fish consumption, and quintiles of total food consumption (estimated by summing all food items in the food frequency questionnaire).	Total fruit: OR0.9; 95% CI: 0.7-1.2;
Terry et al., 1999 (49)	Sweden	Cohort	11,659 (133 cases)	≥18y	NA	Age, physical activity, weight at enrollment and parity.	Vegetable: RR:0.86;95%CI:0.64-1.10.
Terry et al.,2002b (50)	Sweden	Case-control	1204 cases 1222 controls	50-74y	NA	Age, body mass index, smoking, leisure time physical activity, consumption of alcohol, multivitamin use, and prevalence of diabetes	Total fish: OR0.8; 95% CI: 0.6-1.0.
Tzonou et al.,1996 (51)	Greece	Case-control	145 cases 298 controls	≥18y	NA	Age, schooling years, age at menopause, age at menarche, number of live born children, number of miscarriages, number of abortions, history of oral contraceptive use, history of use of menopausal estrogens, smoking, alcohol intake, coffee drinking, height, body mass index, and energy intake.	Vegetables: OR 0.85; 95% CI: 0.66-1.11; Fruits: OR 0.96; 95% CI: 0.76-1.21.
Xu et al.,2006 (52)	China	Case-control	1204 cases 1222 controls	30-69y	NA	Age, menopausal status, diagnosis of diabetes, alcohol consumption, BMI, physical activity, and total energy intake	Total fish: OR2.4; 95% CI:1.8-3.1.

BMI, Body mass index; HT, Hormone therapy; USA, United states; UK, United Kingdom; AHEI, Alternative healthy eating index; aMED, Alternate Mediterranean diet; DASH, Dietary approaches to stop hypertension; hPDI, Healthy plant-based diet; LCD, Low carbohydrate diet; MIND, Mediterranean-DASH diet intervention for neurodegenerative delay diet; HLS, Healthy lifestyle score; DPI, Dietary phytochemical index. OC,oral contraceptives.

### Healthy dietary patterns and risk of endometrial cancer

**Figure 2:**
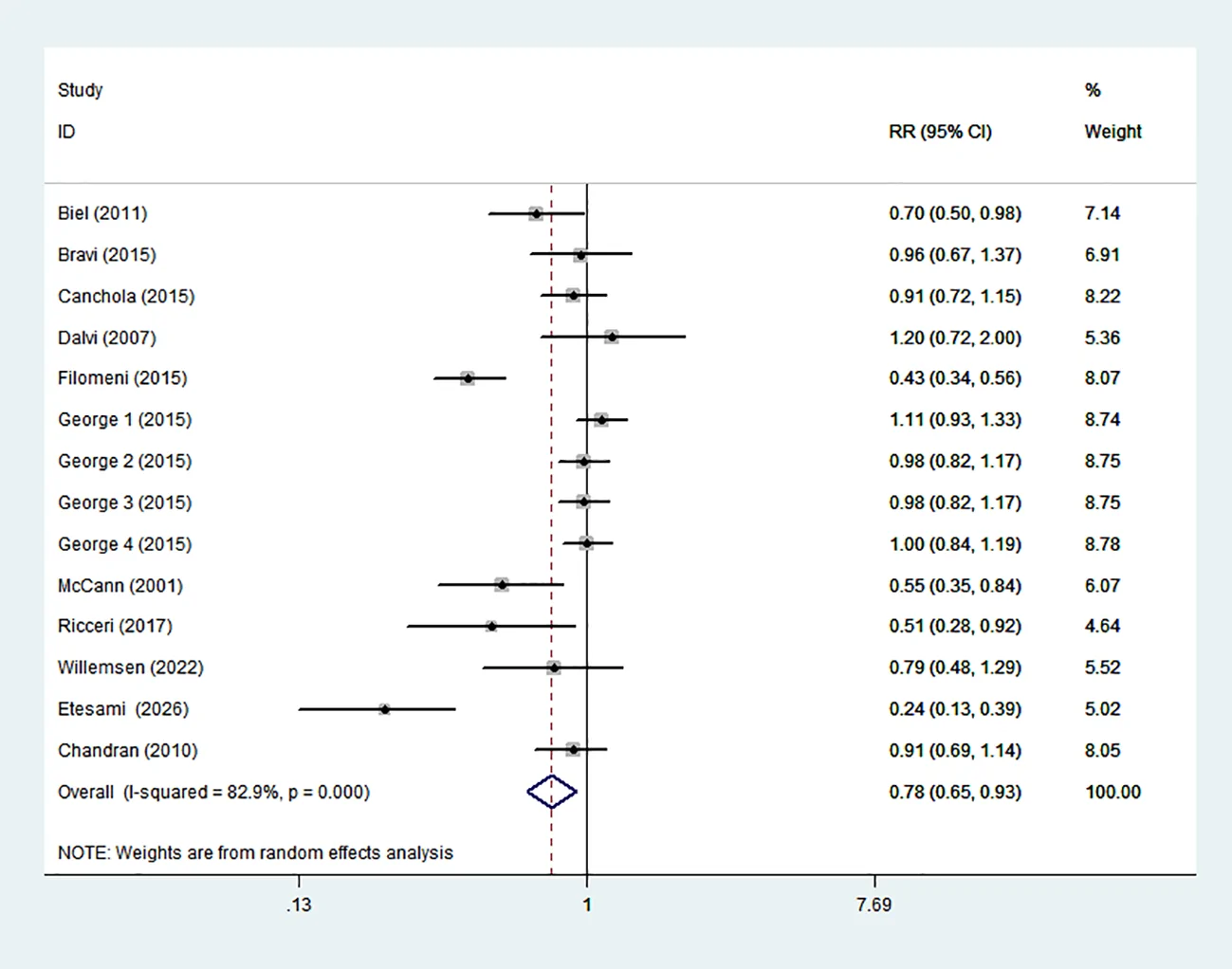
Healthy dietary patterns and risk of endometrial cancer.

Combining 14 effect sizes from eleven articles, including 187,403 participants and 6,415 endometrial cancer cases, revealed that the highest versus lowest adherence to healthy dietary patterns was significantly associated with a 22% reduction in endometrial cancer risk (RR = 0.78; 95%CI:0.65-0.93, *P* = 0.006, **Figure 2**). Significant heterogeneity was observed among the included studies (I^2^ = 82.9%, *P* < 0.001), and therefore a random-effects model was used.

### Typical healthy foods and risk of endometrial cancer

**Figure 3:**
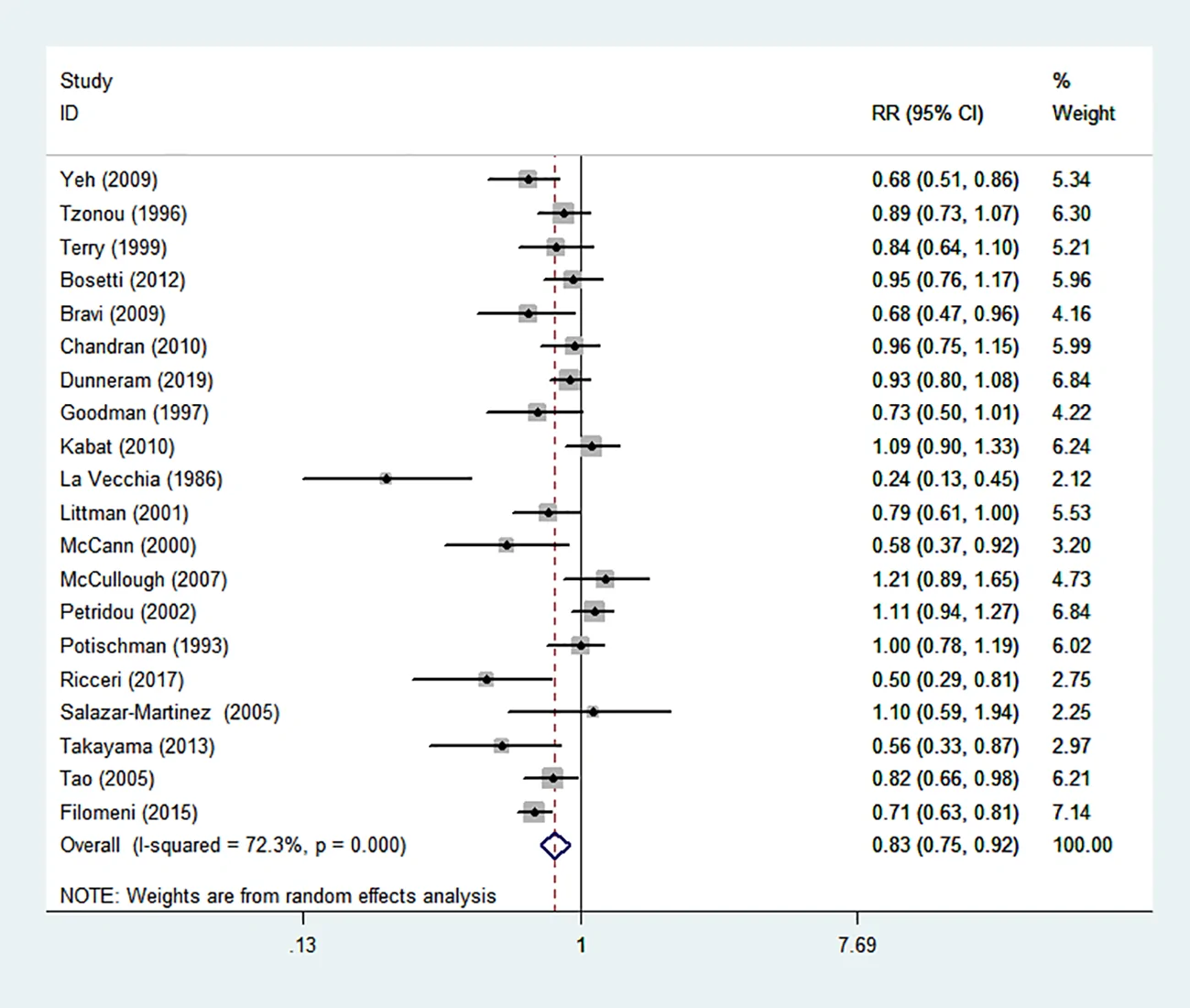
Total vegetables intake and risk of endometrial cancer.

**Figure 4:**
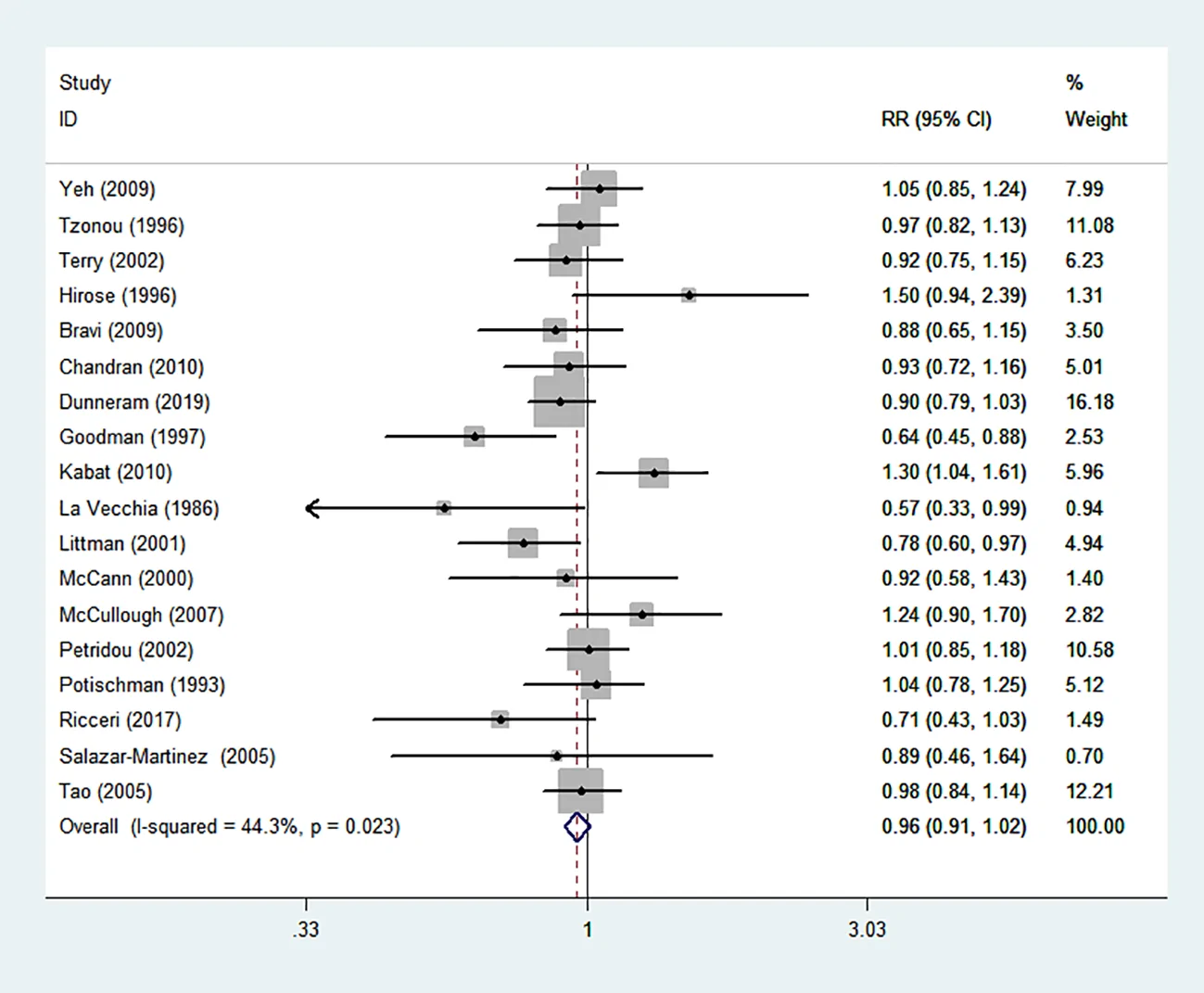
Fruits intake and risk of endometrial cancer.

**Figure 5:**
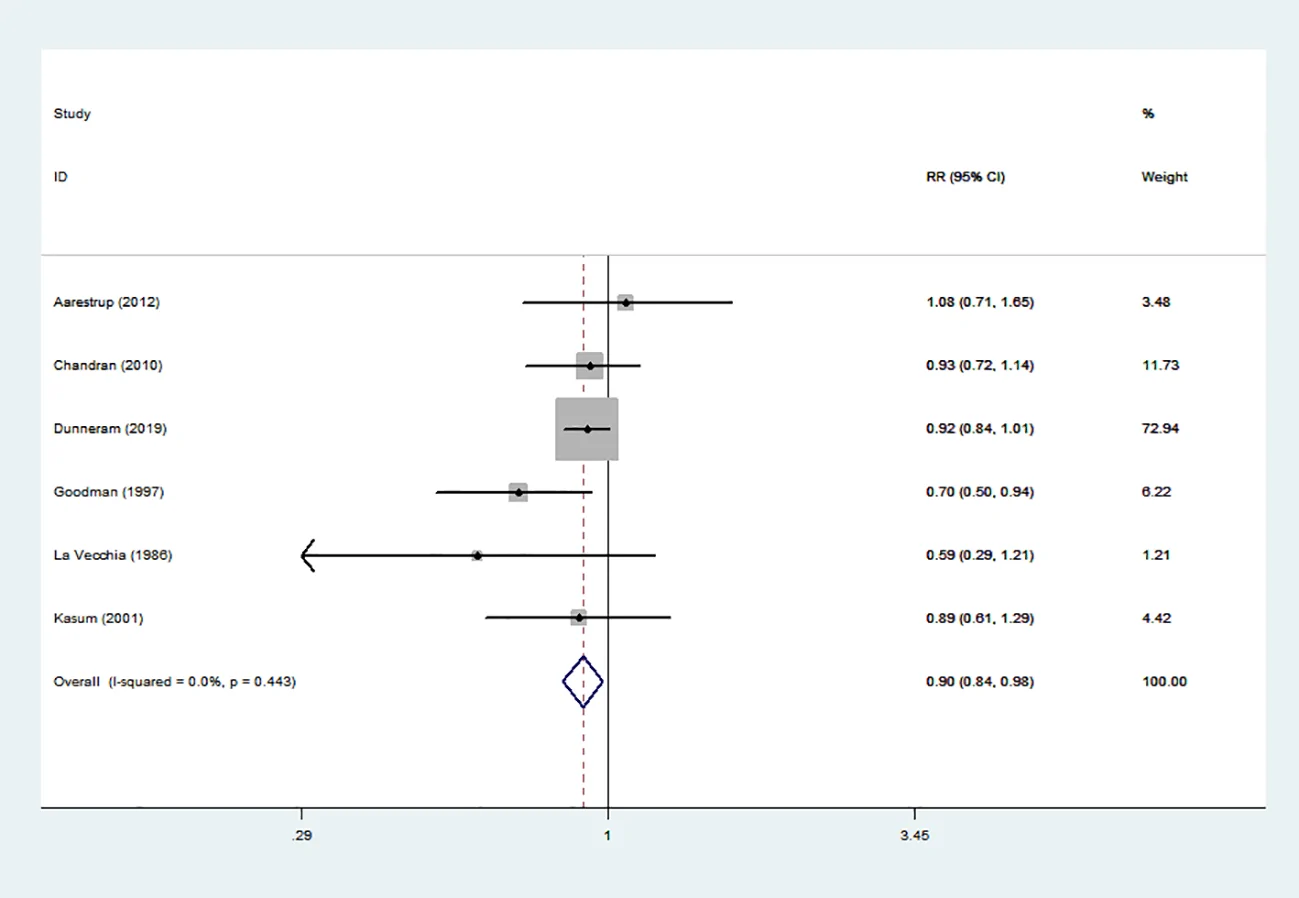
Whole grains and risk of endometrial cancer.

**Figure 6:**
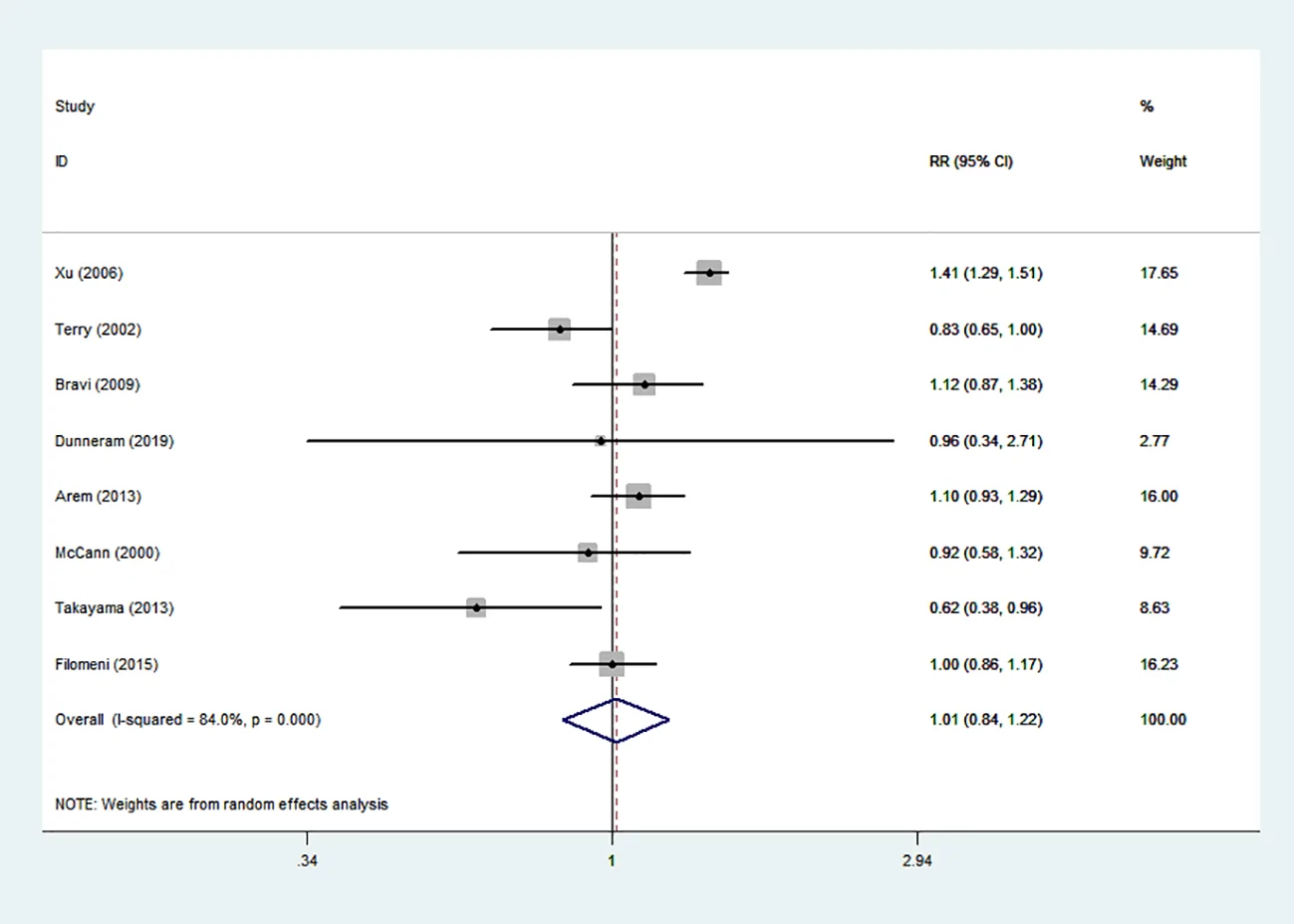
Fish intake and risk of endometrial cancer.

The associations between high intakes of typical healthy foods and endometrial cancer risk are shown in **Figures 3**-**6**. Pooled results form nineteen articles showed an inverse association between total vegetable intake and endometrial cancer risk(RR = 0.83; 95%CI:0.75-0.92; *P* = 0.001) (**Figure 3**). Significant heterogeneity was present (I^2^ = 72.3%, *P* < 0.001), and therefore the effect size was assessed using a random-effects model. In contrast, pooled results form eighteen articles showed that high fruit intake was not associated with endometrial cancer risk (RR = 0.96; 95%CI: 0.91-1.02; *P* = 0.161) (**Figure 4**). A fixed-effects model was used, with low heterogeneity (I^2^ = 44.3%, *P* = 0.023). As shown in **Figure 5**, the highest versus lowest intake of whole grains was associated with a reduced risk of endometrial cancer (RR = 0.90; 95%CI:0.84-0.98; *P* = 0.012), with no evidence of heterogeneity (I^2^ = 0.0, *P* = 0.443). A fixed-effects model was used. Moreover, pooled results form eight articles showed that high fish intake was not associated with endometrial cancer risk (RR = 1.01; 95%CI:0.84-1.22; *P* = 0.882), and there was significant heterogeneity (I^2^ = 84.0%, *P* < 0.001) (**Figure 6**).

### Subgroup analyses

Given the significant heterogeneity observed in the associations between healthy dietary patterns, vegetables and fish intake and endometrial cancer risk, subgroup analyses were performed to explore potential sources of heterogeneity. For healthy dietary patterns (**Table 2**), subgroup analyses were stratified by study design, methods used to determine healthy dietary patterns, country, study quality, comparison, sample and case sizes. The results demonstrated that healthy dietary patterns were significantly associated with a reduced risk of endometrial cancer in studies that used a posteriori method to identify healthy dietary patterns (RR = 0.79, 95%CI:0.65-0.96), with low heterogeneity (I^2^ = 40.9%, *P=*0.119). Moreover, in studies using Q4 vs Q1 or T3 vs T1 comparisons, significant heterogeneity was present (I^2^ = 75.1%, *P* = 0.001), and a decreased risk of endometrial cancer was observed (RR = 0.64; 95%CI: 0.47-0.87). For sample size, subgroup analysis also showed an inverse association between healthy dietary patterns and risk of endometrial cancer in studies with sample size<5000 (RR = 0.67; 95% CI:0.48-0.93), with significant heterogeneity (I^2^ = 78.3%, *P<*0.001).

**Table 2:** Subgroup analyses of healthy dietary patterns and risk of endometrial cancer.

Dietary patterns	Subgroup	Number	RR(95%CI)	I^2^(%)	*P*_for heterogeneity_
Healthy dietary patterns	**Study design**				
	Case-control	8	0.63(0.46-0.87)	83.2	<0.001
	Cohort	3	1.00(0.92-1.08)	0.0	0.717
	**Methods used to determine dietary pattern**				
	Priori	4	0.77(0.59-1.00)	90.7	<0.001
	Posteriori	7	0.79(0.65-0.96)	40.9	0.119
	**Country**				
	United States	5	0.97(0.88-1.07)	29.5	0.193
	Other countries	6	0.56(0.39-0.81)	80.6	<0.001
	**Study quality**				
	≥7	9	0.83(0.70-0.98)	79.7	<0.001
	<7	2	0.49(0.13-1.90	94.2	<0.001
	**Comparison**				
	Q5 vs Q1	4	0.90(0.72-1.12)	86.0	<0.001
	Q4 vs Q1/T3 vs T1	7	0.64(0.47-0.87)	75.1	0.001
	**Sample size**				
	>5000	4	0.87(0.70-1.07)	85.8	<0.001
	<5000	7	0.67(0.48-0.93)	78.3	<0.001
	**Case size**				
	<1000	9	0.72(0.56-0.92)	73.0	<0.001
	>1000	2	0.86(0.66-1.14)	90.4	<0.001

Q5, Quintile 5; Q1, Quintile 1; Q4, Quartile 4; Q1, Quartile 1; T3, Tertile 3; T1, Tertile 1.

With regard to typical healthy foods intake (**Table 3**), subgroup analyses were stratified by study design, country, study quality, sample size and comparison. Results showed that vegetable intake was associated with a reduced risk of endometrial cancer in studies with study quality ≥7, Q5 vs. Q1 or T3 vs T1 comparison and using FFQs. Specifically, in studies with study quality ≥7, vegetable intake was associated with a reduced risk of endometrial cancer (RR = 0.82; 95%CI: 0.74- 0.91), with significant heterogeneity (*P=*0.001; I^2^ = 60.8%). In Q5 vs. Q1 or T3 vs T1 comparison studies, significant heterogeneity was observed (*P* < 0.001, I^2^ = 73.0%), and a slightly decreased risk of endometrial cancer was shown (RR = 0.73; 95%CI: 0.60- 0.88). Similarly, in subgroup analyses stratified by dietary assessment method, an inverse association was observed in studies using FFQs (RR = 0.87; 95%CI: 0.78-0.97), with significant heterogeneity (*P<*0.001; I^2^ = 70.6%).

**Table 3:** Subgroup analyses of typical healthy foods and risk of endometrial cancer.

Dietary factors	Subgroup	Number	RR (95%CI)	I^2^(%)	*P*_for heterogeneity_
Vegetables	**Study design**				
	Case-control	16	0.78(0.69-0.89)	73.2	<0.001
	Cohort	4	0.99(0.87-1.14)	35.2	0.201
	**Country**				
	Western countries	17	0.83(0.74-0.94)	75.2	<0.001
	Asian countries	3	0.79(0.59-1.05)	38.3	0.198
	**Study quality**				
	≥7	16	0.82(0.74-0.91)	60.8	0.001
	<7	4	0.81(0.59-1.12)	87.2	<0.001
	**Sample size**				
	≥5000	5	0.92(0.76-1.12)	80.3	<0.001
	<5000	15	0.79(0.69-0.90)	71.0	<0.001
	**Comparison**				
	Q4 vs Q1	10	0.91(0.83-1.01)	55.7	0.016
	Q5 vs Q1/T3 vs T1	10	0.73(0.60-0.88)	73.0	<0.001
	**Dietary assessment method**				
	FFQ	15	0.87(0.78-0.97)	70.6	<0.001
	Other	5	0.66(0.48-0.90)	77.4	0.001
Fish	**Study design**				
	Case-control	6	0.99(0.78-1.25)	88.2	<0.001
	Cohort	2	1.10(0.93-1.29)	0.0	0.800
	**Country**				
	Western countries	6	1.01(0.92-1.11)	4.4	0.388
	Asian countries	2	0.97(0.43-2.16)	91.5	0.001
	**Sample size**				
	<5000	5	0.98(0.73-1.32)	88.4	<0.001
	≥5000	3	1.04(0.93-1.17)	0.0	0.699
	**Comparison**				
	Q4 vs Q1	4	1.04(0.71-1.51)	87.4	<0.001
	Q5 vs Q1/T3 vs T1	4	1.02(0.87-1.18)	48.9	0.118
	**Dietary assessment method**				
	FFQ	6	1.08(0.88-1.31)	82.9	0.001
	Other	2	0.85(0.70-1.03)	0.0	0.664

FFQ, Food, frequency questionnaire; Q5, Quintile 5; Q1, Quintile 1; Q4, Quartile 4; Q1, Quartile 1; T3, Tertile 3; T1, Tertile 1.

### Publication bias

Visual inspection of funnel plots (**Supplementary Figure 1**) and asymmetry tests showed evidence of publication bias for healthy dietary patterns (Begg’s test *P* = 0.025; Egger’s test *P* = 0.044). We therefore used the trim-and-fill method to re-estimate the pooled RRs (**Supplementary Figure 2**). The results showed that no new studies were imputed to the funnel plot, and the overall RR remained unchanged (RR = 0.780; 95%CI:0.653-0.932, *P* < 0.001). Similarly, as shown in **Supplementary Figure 3**, visual inspection of the funnel plot for total vegetable intake revealed some degree of publication bias. Begg’s test also showed publication bias for the association between total vegetable intake and endometrial cancer risk (Begg’s test *P* = 0.009).Therefore, we used the trim-and-fill method to re-estimate the pooled RRs (**Supplementary Figure 4**). The results showed that no studies were imputed to the funnel plot, and the overall RR remained unchanged (RR = 0.830;95%CI:0.747-0.922, *P* < 0.001). For fish intake, due to the relatively small number of included studies, publication bias could no be conducted to explore potential sources of heterogeneity.

### Sensitivity analysis

As shown in **Supplementary Figures 5**-**7**, the associations between healthy dietary patterns, total vegetable intake and whole grain intake and endometrial cancer risk were robust and not affected by any single study or a couple of studies. However, as shown in **Supplementary Figure 8**, the study by Xu et al. (52) exceeded the range and may have been a potential source of heterogeneity. After excluding this study in a repeat analysis, the pooled RR for the association between fish intake and endometrial cancer risk showed a slight decrease(RR = 0.97; 95%CI: 0.86-1.10, *P* = 0.667) (**Supplementary Figure 9**). Moreover, heterogeneity decreased from 84.0 to 35.6%.

### Quality assessment

The methodological quality of the included non-randomized studies based on NOS criteria is shown in **Table 4**. Studies with a NOS score of ≥7 were considered to be of relatively high quality (6–8, 10, 13, 14, 17–23, 33–39, 41, 42, 45–50, 52), while the remaining six articles were identified as medium quality (15, 16, 40, 43, 44, 51).

**Table 4:** Healthy dietary patterns, typical healthy foods and endometrial cancer: assessment of study quality.

Studies	Selection				Comparability		Outcome			
	1	2	3	4	5A	5B	6	7	8	Score
Cohort										
Dunneram et al., 2019 (7)	*	*	*	*	*	*	*	*	*	9
McCullough et al., 2007 (10)	*	*	*	*	*		*	*	*	8
Canchola et al., 2015 (17)	*	*		*	*	*	*	*		8
George et al., 2015 (19)	*	*	*	*	*	*	*	*	*	9
Willemsen et al., 2022 (22)	*	*	*	*	*	*	*	*	*	9
Aarestrup et al.,2012 (33)	*	*	*	*	*		*	*	*	8
Arem et al., 2013 (34)	*	*	*	*	*	*	*	*	*	9
Kabat et al.,2010 (38)	*	*	*	*	*	*	*	*	*	9
Kasum et al.,2001 (39)	*	*	*	*	*		*	*	*	8
Terry et al., 1999 (49)	*	*	*	*	*		*	*	*	8
Case-control										
Yeh et al., 2009 (6)	*	*	*	*	*		*	*		7
Goodman et al., 1997 (8)	*	*	*	*	*		*	*		7
Dalvi et al., 2007 (13)	*	*	*		*	*	*	*		7
Biel et al., 2011 (14)	*	*	*	*	*		*	*		7
Etesami et al.,2026 (15)	*	*	*		*		*	*		6
Bravi et al., 2015 (16)	*	*	*		*		*	*		6
Filomeni et al., 2015 (18)	*	*	*	*	*		*	*		7
McCann et al., 2001 (20)	*	*	*	*	*		*	*		7
Ricceri et al., 2017 (21)	*	*	*	*	*		*	*		7
Chandran et al., 2010 (23)	*	*	*	*	*		*	*		7
Bosetti et al., 2012 (35)	*	*	*	*	*		*	*		7
Bravi et al., 2009 (36)	*	*	*	*	*		*	*		7
Hirose et al.,1996 (37)	*	*	*	*	*		*	*		7
La Vecchia et al., 1986 (40)	*	*	*		*		*	*		6
Littman et al., 2001 (41)	*	*	*	*	*		*	*		7
McCann et al., 2000 (42)	*	*	*	*	*		*	*		7
Petridou et al., 2002 (43)	*	*	*		*		*	*		6
Potischman et al., 1993 (44)	*	*	*		*		*	*		6
Salazar-Martinez et al., 2005 (45)	*	*	*	*	*		*	*	*	8
Takayama et al., 2013 (46)	*	*	*	*	*		*	*		7
Tao et al.,2005 (47)	*	*	*	*	*		*	*		7
Tzonou et al.,1996 (51)	*	*	*		*		*	*		6
Terry et al.,2002a (48)	*	*	*	*	*		*	*	*	8
Terry et al.,2002b (50)	*	*	*	*	*		*	*		7
Xu et al.,2006 (52)	*	*	*	*	*		*	*		7

*For case-control studies, 1 indicates cases independently validated; 2, cases are representative of population; 3, community controls; 4, controls have no history of endometrial cancer; 5A, study controls for age; 5B, study controls for additional factor(s); 6, ascertainment of exposure by blinded interview or record; 7, same method of ascertainment used for cases and controls; and 8, non-response rate the same for cases and controls. For cohort studies, 1 indicates exposed cohort truly representative; 2, non-exposed cohort drawn from the same community; 3, ascertainment of exposure; 4, outcome of interest not present at start; 5A, cohorts comparable on basis of age; 5B, cohorts comparable on other factor(s); 6, quality of outcome assessment; 7, follow-up long enough for outcomes to occur; and 8, complete accounting for cohorts.

## Discussion

To our knowledge, this is the largest and most comprehensive systematic review and meta-analysis specifically assessing the associations between healthy dietary patterns, typical healthy foods and endometrial cancer risk. Incorporated thirty-five articles with aggregate data on 533,828 participants and 17,758 endometrial cancer cases, our findings revealed that higher intakes of healthy dietary patterns, vegetables and whole grains were significantly associated with a lower risk of endometrial cancer, whereas higher intakes of fruit and fish were not associated with the risk of endometrial cancer. Collectively, our findings provide strong evidence for an inverse association between healthy dietary patterns and endometrial cancer risk, supporting the adoption of this pattern for endometrial cancer prevention. However, the absence of a dose-response analysis warrants caution in the interpretation of these results.

### Comparison with epidemiological studies

Given the growing global burden of endometrial cancer, identifying modifiable risk factors for early prevention is an urgent public health priority. Dietary patterns, accounting for combinations of various food groups and beverages consumed as part of the whole diet, have been used to evaluate the association between diet and endometrial cancer. Over the years, the associations between healthy dietary patterns and endometrial cancer risk have been widely studied, albeit with inconsistent results (13–23). For instance, in a hospital-based case-control study, Etesami et al. found that higher adherence to the Healthy Eating Index (HEI)-2020 was associated with a reduced risk of endometrial cancer among Iranian women (15). Similarly, Biel et al. also found that a plant-based pattern was significantly associated with a reduced risk of endometrial cancer in a population-based case-control study in Canada (14). In contrast, Canchola et al. found that a plant-based dietary pattern was not associated with endometrial cancer risk in the California Teachers Study cohort (17). Consistent with these studies, we also found a significant inverse association between adherence to healthy dietary patterns and endometrial cancer risk, despite the presence of significant heterogeneity. This inconsistency across studies may be attributable to differences in study design, sample size, dietary habits and the methods used to identify dietary patterns.

Although evidence regarding the association between healthy dietary patterns and endometrial cancer risk remains inconclusive, several potential mechanisms may explain the observed beneficial effect. First, vegetables and fruits, major components of healthy dietary patterns, are rich sources of antioxidants, such as vitamin C, polyphenols and carotenoids. Previous studies have shown that these antioxidants can neutralize reactive oxygen species and protect against free radicals-induced damage involved in carcinogenesis (53, 54). Second, vegetables, fruits, legumes, and whole grains are good sources of dietary fiber. Higher dietary fiber intake has been significantly associated with a lower risk of type 2 diabetes mellitus (55), a well-established risk factor for endometrial cancer (4). In addition, studies have shown that high dietary fiber intake can regulate plasma sex hormones concentrations, including reducing serum levels of estrone and 17β-estradiol (56). Third, vegetables and fruits are rich in folic acid, which is essential for thymine synthesis and plays a crucial role in DNA synthesis, repair and methylation, thereby protecting against carcinogenesis (57). Finally, the beneficial effect of adherence to healthy dietary patterns on endometrial cancer may be partly attributed to lower intakes of unhealthy foods, such as red meat, processed meat, and sweets. A previous systematic review and meta-analysis showed that a Western/unhealthy dietary pattern, characterized by high intakes of red and processed meat, animal fat, salty snacks, refined grains, sweets, and fast food, was significantly associated with a higher risk of endometrial cancer (24).Taken together, these mechanisms support our conclusion that healthy dietary patterns are associated with a protective effect against endometrial cancer.

The findings of this meta-analysis also revealed an inverse association between total vegetable intake and endometrial cancer risk. Our study findings are in consistent with a previous meta-analysis of observational studies by Lu et al. (9), which demonstrated that vegetable consumption was significantly associated with a reduced risk of endometrial cancer. Several plausible mechanisms may explain the protective effect of vegetable intake on endometrial cancer risk. The beneficial effects of vegetables may be attributed to their content of vitamins, flavonoids, phytoestrogens, and other micronutrients, which are known to have antioxidant and anticancer properties. As discussed above, vegetables are good sources of folic acid, dietary fiber, and antioxidants, such as vitamin C and carotenoids. Antioxidants can neutralize reactive oxygen species and protect against free radical induced damage involved in carcinogenesis (53, 54). A previous dose-response meta-analysis of nine studies assessing dietary vitamin C intake reported a protective effect of higher vitamin C intake on endometrial cancer risk (58). Moreover, dark green vegetables are rich sources of folate. Davis and colleagues have confirmed that folate is essential for thymine synthesis and plays a critical role in DNA synthesis, repair, and methylation, thereby protecting against carcinogenesis (57). Finally, previous studies have shown that high intake of dietary fiber, an important component of vegetables, is associated with a lower risk of endometrial cancer (59). Collectively, these mechanisms may explain the beneficial association between total vegetable intake and endometrial cancer risk.

In our analyses, high intake of whole grains was associated with a reduced risk of endometrial cancer. Contrary to our findings, previous cohort studies reported no significant association between whole grains and endometrial cancer risk (7, 33, 39). This discrepancy may be partly attributable to variability in study design, dietary assessment methods, and sample size. Although the underlying mechanisms have not been fully elucidated, several mechanisms may support the observed inverse association between whole grain intake and endometrial cancer risk. First, the protective effect may be explained by the high content of dietary fiber. A previous meta-analysis showed that high dietary fiber intake was associated with a 29% reduced risk of endometrial cancer (59). Second, whole grains have a relatively low glycemic index, and evidence suggests that low glycemic index was associated with a lower risk of diabetes mellitus, an important risk factor for endometrial cancer (60). Third, dietary fiber may reduce circulating levels of endogenous bioavailable estrogen by increasing sex hormone-binding globulin levels and interfering with enterohepatic circulation (61).

In this meta-analysis, we found that high fruit intake was not associated with the risk of endometrial cancer. Our results are consistent with two previous cohort studies (7, 10), which also found no evidence of an association between fruit intake and endometrial cancer. The absence of a significant association in prospective studies suggests that recall or selection bias may have influenced the findings in case-control studies. Notably, to our knowledge, previous studies on fruit intake and endometrial cancer risk have been predominantly case-control in design (6, 8, 21, 23, 36, 40–45, 47, 48, 51), and these studies varied considerably in sample size, study population, dietary assessment methods, analytical methods, and presentation of results. In contrast to our findings, a previous meta-analysis of observational studies reported a significant inverse association between fruit intake and endometrial cancer risk (OR = 0.81, 95%CI: 0.70-0.94) (9).This discrepancy may be explained by the inclusion of a larger number of studies in our analysis. In addition, we excluded the study by Esposito et al., which was included in the meta-analysis by Lu et al. (9). Consequently, compared with the earlier meta-analysis, our study included more studies and has a larger sample size, providing sufficient statistical power to estimate the true association between fruit intake and endometrial cancer risk.

No statistically significant association was found between fish intake and endometrial cancer risk in our study (*P*>0.05). Similarly, previous cohort studies also reported no association between fish intake and endometrial cancer risk (7, 34). To date, however, studies on this topic remain limited and have yielded inconsistent findings. For example, Takayama et al. reported that higher fish intake was associated with a reduced risk of endometrial endometrioid adenocarcinoma in Japanese women (46), whereas in a hospital-based case-control study by Bravi et al., high fish intake was not associated with endometrial cancer risk (36). Several factors may explain the observed null association. First, the available evidence is limited. Only eight English-language articles were included in this meta-analysis, and studies published in other languages were excluded. Second, the majority of included studies were case-control in design, which are susceptible to recall and selection biases, thereby affecting the results. Third, the inconsistent findings across studies may reflect differences in different level of fish intake. Fourth, in all included studies, adjustment for potential confounding variables were inconsistent in multivariate models, which may explain the null result observed in our analyses. In summary, more high-quality prospective cohort studies are needed to further clarify the relationship between fish intake and endometrial cancer risk.

In this meta-analysis, significant between-study heterogeneity was found between healthy dietary patterns and endometrial cancer (I^2^ = 82.9%, *P* < 0.001). As is common in previous meta-analyses (5, 27), such heterogeneity is not unexpected. To explore the potential sources of significant heterogeneity, we carried out subgroup analyses based on study design, methods used to determine healthy dietary patterns, country, study quality, comparison, sample and case sizes. The results showed that significant heterogeneity was largely attributable to differences in the methods used to determine dietary patterns, comparison, and sample size. For instance, when analyses were stratified by methods used to determine dietary patterns, heterogeneity decreased from 82.9% to 40.9%. Although the sources of heterogeneity could not be fully explained, several factors may have contributed. First, variation in adjustment for potential confounders across the multivariate models used in included studies may have contributed to the observed heterogeneity. Second, significant heterogeneity may be attributed to the difficulty in characterizing healthy dietary patterns across diverse populations (62). Third, although all studies used the highest versus lowest category for comparison (taking the lowest as the reference), the scoring ranges were divided into different intervals across studies, which may have contributed to heterogeneity. Fourth, the majority of included studies were case-control in design, which are susceptible to recall and selection biases. Ultimately, residual heterogeneity remained in subgroup analyses, suggesting that other unmeasured factors may also have contributed.

### Strengths and limitations

This study has several strengths. First, this is the latest and most comprehensive systematic review and meta-analysis assessing the associations between healthy dietary patterns, typical healthy foods, and endometrial cancer risk. Compared with previous meta-analyses (5, 24), we not only provide timely updates, but also include more original articles with larger numbers of participants and endometrial cancer cases, thereby providing more robust evidence. Our findings add to the further evidence supporting a beneficial association between higher adherence to healthy dietary patterns and endometrial cancer risk, underscoring the importance of this pattern in the prevention of endometrial cancer. Second, endometrial cancer cases in the included studies were identified through medical records, avoiding misdiagnosis bias. Third, we strictly followed the predetermined inclusion and exclusion criteria when screening the literature. Fourth, subgroup and sensitivity analyses were conducted to identify potential sources of heterogeneity, thereby enhancing the robustness of the research results. Finally, all included studies were of moderate to high quality, and the RRs or ORs were adjusted estimates from multivariate analyses. Despite these strengths, several important limitations should be mentioned. First, the observational nature of all eligible studies precludes causal inference regarding the associations between healthy dietary patterns, typical healthy foods, and endometrial cancer risk. Second, the vast majority of included studies used FFQs to collect dietary information, which are subject to inherent recall bias. In addition, dietary assessment was subjectively measured, which may introduce measurement error and potentially attenuate the observed associations. Third, although numerous potential confounders have been adjusted for, residual confounding by certain unmeasured factors cannot be completely ruled out. Fourth, due to insufficient data in the included studies, we were unable to perform dose-response analyses, which precluded quantification of the associations between healthy dietary patterns, typical healthy foods, and endometrial cancer. Fifth, significant heterogeneity existed in the main analysis. Although subgroup and sensitivity analyses were performed to explore possible sources of heterogeneity, we were unable to fully explain the inter-study heterogeneity. Sixth, significant publication bias was detected in healthy dietary patterns and total vegetable intake. However, after applying the trim-and-fill method, no studies were imputed, and the results remained unchanged. Seventh, the restriction to studies published in English or Chinese may have introduced language bias, potentially excluding relevant studies published in other languages (e.g., Korean, or French). Finally, most included studies were performed in developed countries, with only four from developing countries, limiting the generalizability of our findings to other populations. In addition, we acknowledge that the inclusion of CNKI as the sole regional database in our primary search may also have limited the geographical representativeness of the evidence base.

## Conclusion

In conclusion, this study found that higher adherence to healthy dietary patterns was associated with a lower risk of endometrial cancer. In addition, higher intakes of typical healthy foods, including vegetables and whole grains were inversely associated with endometrial cancer risk. Our findings strengthen the existing evidence supporting the beneficial role of healthy dietary patterns and typical healthy foods in relation to endometrial cancer, and highlight the importance of promoting such dietary patterns and increasing the intakes of these foods for endometrial cancer prevention. Nevertheless, due to the absence of dose-response data and presence of significant heterogeneity, these results should be interpreted caution. Further population-based longitudinal studies, particularly in diverse geographic and cultural settings, are needed to corroborate our research findings.

## Data Availability

The original contributions presented in the study are included in the article/**Supplementary Material**. Further inquiries can be directed to the corresponding author.
