# Supplementary material for: Healthy dietary patterns, typical healthy foods and risk of endometrial cancer: a systematic review and meta-analysis of observational studies
Source: Front Oncol. 2026 Aug 12;16:1901081. doi: 10.3389/fonc.2026.1901081 (PMC13506386; doi:10.3389/fonc.2026.1901081)
Supplement: Supplementary file 1 [file Table1.doc]

**Search terms in PubMed, Embase and Web of Science**

(“endometrial cancer”[all fields] OR “endometrial neoplasms”[all fields] OR “endometrial tumor”[all fields] OR “endometrial carcinoma” [all fields]) AND (“diet”[all fields] OR “food”[all fields] OR “dietary pattern” [all fields] OR “food pattern”[all fields])

**Search terms in CNKI**

饮食 AND 子宫内膜癌

**Search terms in other sources**

Reference lists from the retrieved articles and systematic reviews were manually searched to identify relevant studies.
