# Supplementary material for: Healthy dietary patterns, typical healthy foods and risk of endometrial cancer: a systematic review and meta-analysis of observational studies
Source: Front Oncol. 2026 Aug 12;16:1901081. doi: 10.3389/fonc.2026.1901081 (PMC13506386; doi:10.3389/fonc.2026.1901081)
Supplement: Supplementary file 2 [file Table2.doc]

Table 2. The PECOS criteria used for this systematic review and meta-analysis

| **Population** | **Adults** |
| --- | --- |
| Exposure | Healthy dietary patterns and healthy foods |
| Comparison | Highest category vs. lowest category of exposure |
| Outcomes | Endometrial cancer |
| Study design | Cohort and case-control studies |

PECOS: participant, intervention(exposure), comparison, outcome, and study design.
